# Image Registration with Particles, Examplified with the Complex Plasma Laboratory PK-4 on Board the International Space Station

**DOI:** 10.3390/jimaging5030039

**Published:** 2019-03-14

**Authors:** Mierk Schwabe, Milenko Rubin-Zuzic, Christoph Räth, Mikhail Pustylnik

**Affiliations:** Institut für Materialphysik im Weltraum, Deutsches Zentrum für Luft- und Raumfahrt (DLR), Münchener Str. 20, 82234 Weßling, Germany

**Keywords:** image registration, image fusion, complex plasmas, dusty plasmas, particle tracing

## Abstract

Often, in complex plasmas and beyond, images of particles are recorded with a side-by-side camera setup. These images ideally need to be joined to create a large combined image. This is, for instance, the case in the PK-4 Laboratory on board the International Space Station (the next generation of complex plasma laboratories in space). It enables observations of microparticles embedded in an elongated low temperature DC plasma tube. The microparticles acquire charges from the surrounding plasma and interact strongly with each other. A sheet of laser light illuminates the microparticles, and two cameras record the motion of the microparticles inside this laser sheet. The fields of view of these cameras slightly overlap. In this article, we present two methods to combine the associated image pairs into one image, namely the SimpleElastix toolkit based on comparing the mutual information and a method based on detecting the particle positions. We found that the method based on particle positions performs slightly better than that based on the mutual information, and conclude with recommendations for other researchers wanting to solve a related problem.

## 1. Introduction

The Russian–European PK-4 Laboratory [1] on board the International Space Station (ISS) is the latest laboratory to study complex plasmas in microgravity, after the predecessors PKE-Nefedov [2] and PK-3 Plus [3,4,5,6]. Complex plasmas consist of micro- or nanoparticles immersed in a low temperature plasma [7]. The particles acquire charges by collecting ions and electrons from the plasma, and interact strongly with each other. They form a soft condensed matter system where, at least in the case of microparticles, the constituent particles can be observed individually by recording their motion with a fast camera and lenses that resolve the pictures of the illuminated particles [8,9].

In laboratories on Earth, gravity pulls the microparticles towards the plasma sheath, where they are levitated by the strong electric field in that region. This leads to a compression of the particle cloud [10]. Since the same electric field that levitates the microparticles accelerates the ions out of the plasma, a fast ion flux results that disturbs the system [11,12,13].

Under microgravity conditions, however, the microparticles are suspended in the bulk of the discharge, where the conditions are more favorable for studying undisturbed systems. This allows, for instance, investigations of velocity distributions [14], Mach cones [15,16], turbulence [17], waves [18,19,20,21,22], lane formation [23], electrorheological plasmas [22], demixing [22,24], particle charging [25], and crystallization [10,26].

**Figure 1:**
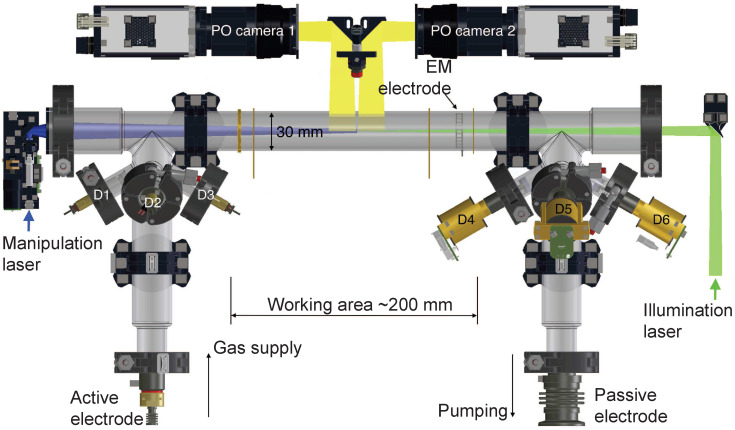
Schematic of the heart of the PK-4 Laboratory. An active and a passive electrode produce a DC plasma inside the glass tube with 30 mm diameter. Six microparticle dispensers (D1–D6) are mounted on the two side tubes. Once injected, the microparticles are transported into the working area with a length of approximately 200 mm in the middle of the plasma chamber. There, they are illuminated by a laser sheet and observed by the two particle observation (PO) cameras. The fields of view (FoV) of the two cameras overlap horizontally by approximately 1.36 mm (not shown in the figure), and are shifted vertically by approximately 0.14 mm. The cameras can be moved along and perpendicular to the tube axis. Reprinted from Pustylnik et al., Rev. Sci. Instr. 87, 093505 (2016) [1], with the permission of AIP Publishing.

The International Space Station (ISS) enables researchers to perform experiments under the condition of long-term microgravity [27]. The PK-4 Laboratory has been hosted in the Columbus module of the ISS since 2014 [1]. The heart of the PK-4 Laboratory consists of an elongated glass tube, in which a DC plasma is ignited. A schematic of the plasma chamber and the associated diagnostics system is shown in Figure 1. Microparticles are injected via dispensers and move into the central area, where a sheet of laser light (shown in green in Figure 1) illuminates them. A manipulation laser, shown in purple, can be used to push the microparticles, producing a central microparticle flow. Two Particle Observation (PO) cameras record the light scattered by the microparticles. Their images have a size of $1600\times 1200\;{\mathrm{px}}^{2}$ each, overlap horizontally by approximately 95 px, and are shifted vertically by approximately 10 px (see below). Selecting the size of the overlap area is a trade-off between two factors: On the one hand, it should be large enough to allow an accurate determination of the transformation between the images recorded with the two cameras. On the other hand, a large overlap area reduces the total field of view. The optimal width of the overlap area thus also depends on the number density of particles and the length of the image sequence considered—the more features/particles the images have, the better the accuracy of joining the images.

The spatial resolutions of the cameras in horizontal (*x* direction, along the tube axis) and vertical (*y* direction, perpendicular to the tube axis) direction, ${\mathrm{res}}_{x}$ and ${\mathrm{res}}_{y}$, respectively, were measured as [1]

(1) $$\begin{array}{cc} \mathrm{Camera}\;1:\; & {\mathrm{res}}_{x}=14.18\pm 0.02\;\mu m/\mathrm{px} \end{array}$$

(2) $$\begin{array}{c} \;\;\;\;\;{\mathrm{res}}_{y}=14.25\pm 0.02\;\mu m/\mathrm{px} \end{array}$$

(3) $$\begin{array}{cc} \mathrm{Camera}\;2:\; & {\mathrm{res}}_{x}=14.20\pm 0.03\;\mu m/\mathrm{px} \end{array}$$

(4) $$\begin{array}{c} \;\;\;\;\;{\mathrm{res}}_{y}=14.31\pm 0.03\;\mu m/\mathrm{px}. \end{array}$$

The recorded images are 8 bit pictures containing 256 gray values. The optical system induces some distortions of the bright regions corresponding to particles, which are stronger for Camera 1 than Camera 2.

**Figure 2:**
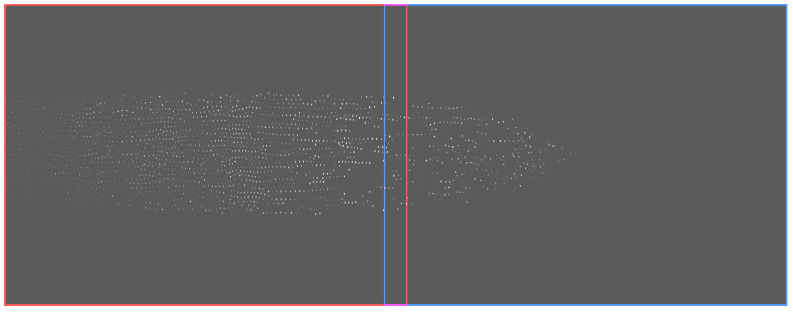
Combined contrast and brightness enhanced images of the two PO cameras. The red frame marks the field of view of Camera 1, the blue one that of Camera 2 (each has a size of $1600\times 1200\;{\mathrm{px}}^{2}$). The pixels in the top right side where no data were recorded were set to black. Combined FoV: $44.1\times 17.1\;{\mathrm{mm}}^{2}$.

To trace particles jointly in both parts of the total field of view, at the very least, the coordinate transform between the spaces defined by the two cameras needs to be known, so that particles can be identified in both sets of images separately and the tracks linked using the coordinate transform. Even better would be to produce joint images, as this allows for more types of particle tracing algorithms and for better visualizations. Figure 2 shows an example of such a joint image with the fields of view of the two cameras indicated by red and blue lines.

The task of overlaying two or more images of the same scene is called “image registration” [28,29,30]. It is a common problem in the fields of remote sensing, medical image processing, and computer vision. This article describes two methods to either join the two images, or to determine a coordinate transform from the coordinate system determined by one field of view to the other, to allow tracing the microparticles in the combined field of view: Method I using the tool kit SimpleElastix based on the mutual information, and Method II based on the particle positions.

On the one hand, we hope that this article proves to be useful to other members of the PK-4 collaboration. On the other hand, we demonstrate the applicability of the widely used image registration tool kit SimpleElastix [31] and compare its output with calculations using the microparticle positions directly. We conclude with concrete recommendations for other researchers facing the same task.

## 2. Image Registration Using SimpleElastix (Method I)

SimpleElastix [31,32] is an open source extension of SimpleITK [33] that includes the elastix C++ library [30,34,35]—a collection of high-performance medical image registration algorithms. While the basic image processing algorithms are written in fast C++, SimpleElastix provides bindings to a variety of languages, among them Python, Java and R. The source code, binaries, and extensive documentation and examples are available online [32].

We used SimpleElastix to register a sequence of 178 images recorded during the commissioning phase of PK-4 [36]. The original images in the sequence were cropped to a central region of 510 px height containing the particle cloud (compare Figure 2). Next, the right-most 90 pixels of images from Camera 1 (shown in red in the following), and the left-most 90 pixels of Camera 2 (shown in blue) were cropped to produce the image sequences of the overlap region. The images from Camera 1 were used as “fixed” images, those from Camera 2 as “moving” images, which means that the space of the resulting combined image is the one defined by Camera 1.

The fixed and moving images for each pair of the sequence were compared using the mutual information. The mutual information is a measure of how much information the image intensity in one image contains about the image intensity in the other image [32]. By maximizing the mutual information, the parameters of the best affine transformation were determined—a transformation that allows only translation, rotation, scaling, and shear.

**Figure 3:**
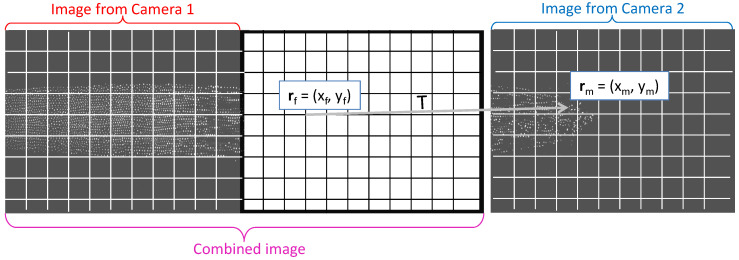
Demonstration of the transformation. We need to fill in the empty pixels of the combined image, shown on the left in this figure. The left part of the combined image is filled by simply copying the image recorded with Camera 1, since the coordinate space for the combined image is that of Camera 1. To fill the right part of the combined image, we need the transformation *T* that will tell us which is the point $r_{m}$ in the right image (recorded with Camera 2) that goes to a certain position $r_{f}$ in the fixed space of the combined image.

The algorithm resulted in six parameters (plus the coordinates of the center of rotation) that allow calculating with subpixel resolution the position $r_{m}=\left(x_{m},y_{m}\right)$ in the moving image that corresponds to each coordinate $r_{f}=\left(x_{f},y_{f}\right)$ in the space of the fixed image. This is demonstrated in Figure 3.

In general, an affine transformation $T(r_{f})$ is given by

(5) $$r_{m}=T\left(r_{f}\right)=A\left(r_{f}-c\right)+t+c,$$

where *A* is the transformation matrix that rotates, scales, and shears, $c=(c_{x},c_{y})$ is the position of the center of rotation, and $t=(t_{x},t_{y})$ is the translation. Using SimpleElastix, we determine $A_{i}$ and $t_{i}$ for each pair of images *i* in the sequence and get the mean values $A={\langle A_{i}\rangle }_{i}$ and $t={\langle t_{i}\rangle }_{i}$

(6) $$\begin{array}{cc} A=\left[\begin{array}{cc} \;\;0.996\pm 0.003 & 0.0064\pm 0.0009\\ -0.0046\pm 0.0008 & 0.9941\pm 0.0004 \end{array}\right] & t=\left[\begin{array}{c} \;\;2.9\pm 0.1\\ -9.50\pm 0.1 \end{array}\right]\mathrm{px}, \end{array}$$

where the uncertainty is the standard deviation of the mean. Please note that the image origin here is in the top left corner of the cropped fixed image, and the center of rotation is the center of the cropped region. To determine the *x*-translation to be applied to the original, non-cropped image, the crop position (x=1510 px) needs to be taken into account.

From our prior measurements, we know that the rotation angle θ is small, so that cosθ≈1 (this assumption is not necessary in general, it just simplifies the further calculations). Furthermore, we assume rotation in clockwise direction and small shear $k_{y}$ only along the vertical direction. Then, the matrix *A* corresponds to

$$A=\left[\begin{array}{cc} s_{x} & s_{y}\left(\sin\theta +k_{y}\right)\\ -s_{x}\sin\theta & s_{y} \end{array}\right],$$

where $s_{x}$ and $s_{y}$ are the scaling factors in horizontal and vertical directions. Comparing with Equation (6), we find

(7) $$\begin{array}{cc} s_{x} & =0.996\pm 0.003 \end{array}$$

(8) $$\begin{array}{cc} s_{y} & =0.9941\pm 0.0004 \end{array}$$

(9) $$\begin{array}{cc} \theta & =-0.264^{\circ }\pm 0.001^{\circ } \end{array}$$

(10) $$\begin{array}{cc} k_{y} & =\left(11\pm 1\right)\times 10^{-3}. \end{array}$$

In [1], 0.9986 for the horizontal scale and 0.9958 for the vertical scale are given, and no rotation is taken into account. Even though the obtained angle of $0.3^{\circ }$ is small, in the resulting images it is non-negligible.

Since we cropped the images at x=1510, the total translation that the moving (right) image should undergo is $t_{\mathrm{mi}}=\left(1510-2.9,9.5\right)=\left(1507.1,9.5\right)$, i.e., translation of $t_{\mathrm{xmi}}=1507.1$ pixels to the right and translation of $t_{\mathrm{ymi}}=9.5$ pixels downwards (please note that the index “mi” indicates that this translation is applied to the coordinates in the moving right image (Camera 2), in contrast to the coordinate transform defined above.)

It is to be noted that SimpleElastix used the pixel centers as origin for the sub-pixel position (see documentation in [32]), whereas other programs might use the left bottom corner of the pixel. Under those conditions, a correction of 0.5 pixels in both directions is necessary.

Next, we wanted to resample the moving image to create a new, combined image in the fixed space. Thus, we created an empty image of size $3110\times 1200\;{\mathrm{px}}^{2}$ (see Figure 3) for each image pair in the sequence. We copied the image from Camera 1 to the left part of the combined image. We chose to join the images at $x_{\mathrm{join}}=1550$ px, which does not correspond to the position of the edge in either image to minimize edge effects.

Then, for each coordinate of the combined image that was not filled by the left image, we calculated the corresponding subpixel position in the space of the moving image and interpolated the intensity values of the surrounding pixels to this target coordinate. Data that would need pixel intensities from coordinates that are out-of-bounds were set to the value 0 in the resulting image. A resulting image is shown in Figure 2.

**Figure 4:**
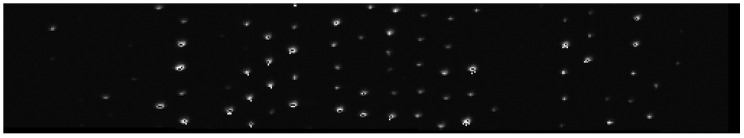
Resulting combined image of the overlap region that is produced by SimpleElastix (image turned by 90${}^{\circ }$ clockwise). Field of view: $7.2\times 1.3\;{\mathrm{mm}}^{2}$.

One way to test the quality of the image registration is to combine the two images of the overlap region. Figure 4 shows an exemplary output automatically created by SimpleElastix. The particles in the overlap region in the left image are more strongly distorted due to the optical system than those in the right image, and thus the overlap of the particles cannot be exact. Nevertheless, Figure 4 clearly shows that the bright regions corresponding to microparticles overlap. Other problems that affect the results of this method are the fact that particles are located at varying transverse positions, which leads to a parallax as seen from the two view points of the cameras, and that the sensitivities of the two cameras are slightly different, so that some particles are overexposed in Camera 1, but not in Camera 2. This leads to an artificial variation in the mutual information and thus errors in the transformation parameters.

## 3. Image Registration Using Detected Particles (Method II)

Since particles are present in the overlap region, the positions of which were used in the further analysis, it seems obvious to use the particle positions to calculate the coordinate transformation. To do so, first the particle positions were detected in the overlap region in all images of the sequence. This was done by a standard method: (1) identify the pixels with brightness lying above a threshold; (2) find the contours of these regions; and (3) either fit a two-dimensional Gaussian to the area within the contour, or, if that fails, use the moment method to calculate the particle position [37]. This resulted in a set of particle positions in the coordinate space of the respective camera.

**Figure 5:**
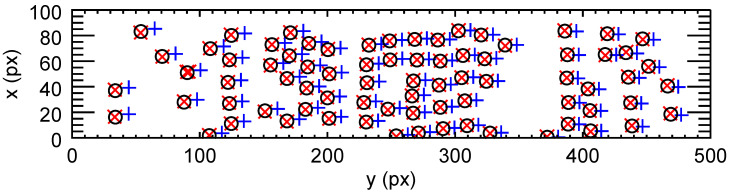
Red crosses: Particle positions in the overlap region in the left image (Camera 1), coordinates given with respect to the bottom left corner of the cropped region. Blue plus signs: Particle positions in the overlap region in the right image (Camera 2), translated only along *x* with the initial guess value $t_{\mathrm{xmi}}$ = −3 px. Black circles: Particle positions from the right image translated with the least squares fit parameters (Equations (11)–(15)).

To determine the transformation between the images, we first needed to select values $t_{\mathrm{xmi}}$ and $t_{\mathrm{ymi}}$ by which to translate the set of particles from the right image, so that the particles in the two images could be identified with each other. For the given sequence, we chose $t_{\mathrm{xmi}}$ = −3 px and $t_{\mathrm{ymi}}$ = −10 px (coordinates are given with respect to the bottom left corner of the cropped image). We then translated the right particles accordingly and searched for a left particle within a circle with a radius of 5 px. If exactly one particle was located within this circle, we assumed that it was the same particle as the one from the other camera, and the particles were associated with each other. Figure 5 shows the resulting particle pairs for one set of images from the sequence.

We then used a least-squares fitting routine [38] to map the positions in Camera 2 to those in Camera 1, assuming an affine transformation (Equation (5)) with no skew, and a rotation by a small angle. This way, we obtained a set of transformation parameters for each image pair. To apply the same transformation to each image pair, we used the mean and standard deviation of these parameters. Converting to the convention used above (origin of *x* and *y* axes in upper left corner), the transformation that gave the position in the moving (right) image that corresponded to a given coordinate in the space of the fixed image has the parameters

(11) $$\begin{array}{cc} s_{x} & =0.998\pm 0.003 \end{array}$$

(12) $$\begin{array}{cc} s_{y} & =0.9945\pm 0.0003 \end{array}$$

(13) $$\begin{array}{cc} \theta & =-0.38^{\circ }\pm 0.03^{\circ } \end{array}$$

(14) $$\begin{array}{cc} t_{x} & =5.6\pm 0.2\;\mathrm{px} \end{array}$$

(15) $$\begin{array}{cc} t_{y} & =-10.5\pm 0.1\;\mathrm{px}. \end{array}$$

These parameters are slightly different from those determined with Method I (Equations (2)–(10)).

## 4. Results

**Figure 6:**
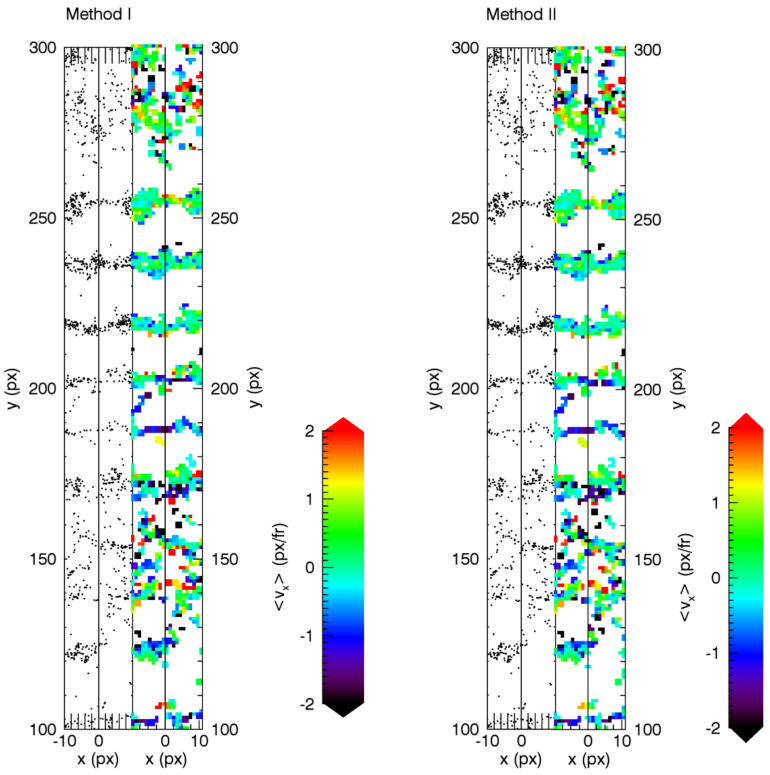
Tracks and average velocities of particles crossing the point (in this plot x=0) where the original left image (Camera 1) was joined to the transformed right image (Camera 2). Even though differences are visible in the positions between the two methods, neither method results in an obvious jump at x=0.

After a visual inspection, we quantified which method produced better results. The resulting combined images were nearly indistinguishable by the naked eye, and the particle tracks in neither showed any significant jumps when they crossed the border where the two images were joined (see Figure 6). Simply plotting the particle positions therefore was not a good method to test the performance of the registration methods.

**Figure 7:**
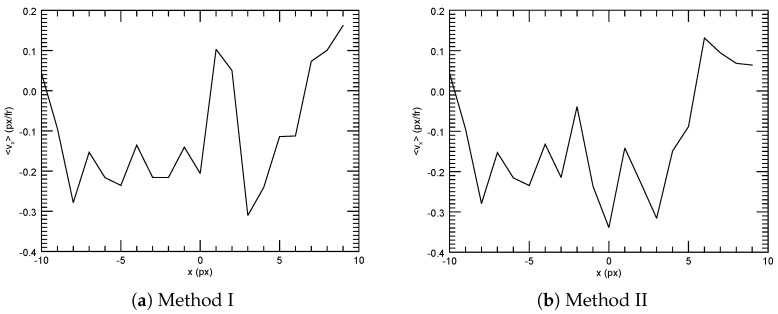
Mean horizontal velocity of traced particles near the point where the two images were joined (x=0 px). A jump of about 0.3 px/fr is visible for Method I.

Figure 7 shows the average horizontal velocity of particles traced across the line where the two images were joined (x=0 px) as a function of *x* position. With both methods we measured a very small horizontal drift of approximately −0.2 px/fr. However, in the plot produced with Method I (Figure 7a), a jump in the mean velocity of approximately 0.3 px/fr after x=0 px is visible, while, in the one from Method II (Figure 7b), the fluctuations after x=0 px are smaller. This is a first indication that Method II might be superior in this case, but it provides no quantitative measure.

**Figure 8:**
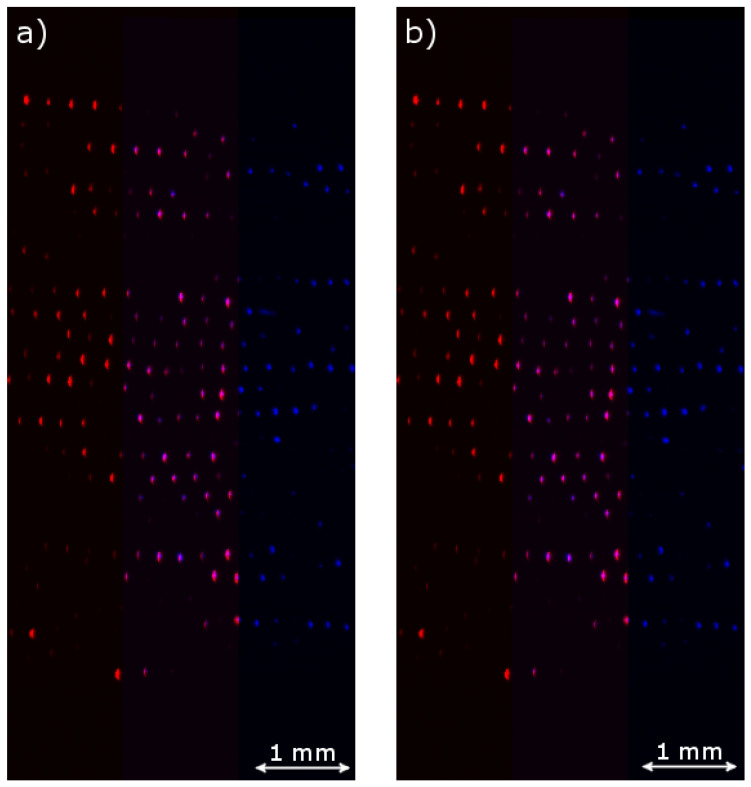
RGB pictures of the region around the overlap. The image from Camera 1 is contained in the red channel, that (after transformation) from Camera 2 in the blue channel. Accordingly, on the left side, only red particles are visible, and, on the right, only blue ones. In the overlap region, the two channels mix, resulting in magenta pixels if both channels contain data. The right image in (**a**) was transformed with Method I (mutual information), that in (**b**) with Method II (particle positions). The left image is identical in (**a**,**b**).

For this purpose, we made color movies of the region around the overlap region encoded in the RGB scheme, inspired by Korolev et al. [39]. These movies show the images of Camera 1 in the red channel and the transformed images of Camera 2 in the blue channel (see Figure 8). The corresponding movies are included in the Supplementary Materials.

Overlapping particles from both cameras then automatically appear in magenta, since both the red and blue color channels of the RGB images contain data. The images of the particles at the right edge of the field of view of Camera 1 were distorted more strongly than in Camera 2, thus no perfectly magenta image was to be expected. However, we could use the number of pixels of this color as an indicator of the quality of the match. We defined as “magenta” those pixels where the difference between the intensity contained in the two channels was (somewhat arbitrarily) less than 50 in value, and we defined as “particle in the overlap region” those pixels where both channels independently had intensity values larger than 20:

magenta pixel = abs(value red channel - value blue channel) < 50

particle in overlap region = value red channel > 20 AND value blue channel > 20

Then, we counted the number of magenta pixels belonging to particles in the overlap region, $n_{\mathrm{mp}}$, in both time series

(16) $$\begin{array}{cc} \mathrm{Method}\;I:\;n_{\mathrm{mp}} & =80844 \end{array}$$

(17) $$\begin{array}{cc} \mathrm{Method}\;\mathrm{II}:\;n_{\mathrm{mp}} & =84633. \end{array}$$

Thus, for this time series, the method based on the particle positions (Method II) produced a 5% better overlap than the method based on the mutual information (Method I).

For our experimental setup, we knew the approximate translation needed to find particle pairs in Method II. If this was not the case, the identification between particles recorded with two side-by-side cameras could be done with a simple method, which we discuss next.

## 5. Automatically Finding the Translation

The particle coordinates can be used for the calculation of the transformation function in Method II only if two requirements are fulfilled:

1. The coordinates of particles in the overlap region that are only seen by one of the cameras have to be removed.
2. The remaining coordinates belonging to the same particles have to be correctly assigned between the coordinate systems of the two cameras.

In Section 4, it is mentioned that a sufficiently accurate estimation of the approximate translation in the overlap region is necessary, after which close particles are determined, and thus the associated coordinates of the same particles can be found.

Since the translation in *x* and *y* is the dominant part of the transformation function, we propose directly associating particle positions seen in the overlap, and removing positions for which no partner is found, by calculating the distances between all particle coordinates in the left and those in the right images. This method only works if enough correct positions of particle pairs are available, ideally in a sequence of images to improve the statistics. We applied the following algorithm:

1. Detection (as described in Section 2) of all particle positions $r_{l}$ and $r_{r}$ in the overlap region of all image pairs in the sequence.
2. For each image pair *i* in the sequence: For all points $r_{l}$, calculate all *x* and *y* distances to each point $r_{r}$, ${\left\{d_{x}\right\}}_{i}$ and ${\left\{d_{y}\right\}}_{i}$.
3. Determination of the “approximate translation” values, $t_{\mathrm{ax}}$ and $t_{\mathrm{ay}}$, as the median values of the distances in all *N* images:
  (18) $$\begin{array}{cc} t_{\mathrm{ax}} & =\mathrm{median}\left\{{\left\{d_{x}\right\}}_{1}\cdots {\left\{d_{x}\right\}}_{N}\right\} \end{array}$$
  (19) $$\begin{array}{cc} t_{\mathrm{ay}} & =\mathrm{median}\left\{{\left\{d_{y}\right\}}_{1}\cdots {\left\{d_{y}\right\}}_{N}\right\}. \end{array}$$
4. For each image pair *i*: Selection of particle pairs in both images where both the *x* and *y* distance is within a certain threshold Δt of the approximate translation (we used Δt=5 px):
  (20) $$\begin{array}{cc} t_{\mathrm{ax}} & -\Delta t<d_{x}<t_{\mathrm{ax}}+\Delta t\;\;\;\mathrm{AND} \end{array}$$
  (21) $$\begin{array}{cc} t_{\mathrm{ay}} & -\Delta t<d_{y}<t_{\mathrm{ay}}+\Delta t. \end{array}$$
  The coordinates that fulfill these conditions belong to the same particle.
  For our image sequence, we found
  (22) $$\begin{array}{cc} t_{\mathrm{ax}} & =1508.8\;\mathrm{px} \end{array}$$
  (23) $$\begin{array}{cc} t_{\mathrm{ay}} & =-8.5\;\mathrm{px}. \end{array}$$

Note that the mean of the distances is not a good measure, since it can be greatly affected by outliers. For our image sequence, we get $\langle \{d_{x,\mathrm{sequence}}\}\rangle =1510.0\;\mathrm{px}$ and $\langle \{d_{y,\mathrm{sequence}}\}\rangle =-2.2\;\mathrm{px}$—the vertical translation would be notably wrong if determined as the mean of the distances.

**Figure 9:**
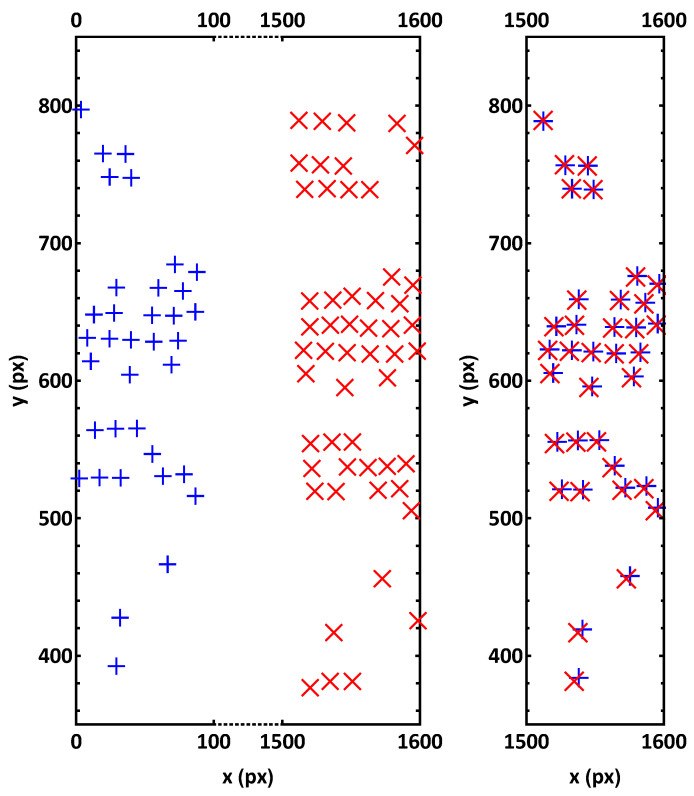
(**Left**) All detected particle coordinates in the overlap region in one image pair recorded by both cameras are shown (all coordinates in the coordinate system of the respective camera). Some coordinates from the left image (red crosses) do not have an associated coordinate in the right image (blue plus signs), and vice versa. (**Right**) The approximate translations, $t_{\mathrm{ax}}=1508.8$ px and $t_{\mathrm{ay}}=-8.5$ px, were used to transform the coordinates of particles with detected partners from the coordinate system of Camera 2 to that of Camera 1 (red crosses and blue plus signs at approximately the same position). Non-associated coordinates (particles seen only by one camera) were removed.

In the left plot of Figure 9, all detected particles in the overlap region of a pair of sample images are seen. Some of the particles in the left image (marked with red crosses) do not have an associated particle in the right image (blue plus signs), and vice versa. Typically, more particles in our sequence are detected by Camera 1 due to its higher sensitivity.

After the described method was adopted, all particles that do not have an appropriately associated partner are successfully removed (see right plot of Figure 8). The remaining particles are simultaneously assigned in pairs, so that the affine transformation (Equation (5)) is directly applicable, and the corresponding values for translation, scaling, rotation and shear between the two camera images can be found.

## 6. Conclusions

In this paper, we present two methods to combine images of particles from two cameras that are mounted parallel to each other. The first method uses the mutual information to find the best affine transformation between the image pair, as implemented in the open source tool kit SimpleElastix [31,32]. The second method depends on first detecting the particle coordinates and then implements a least squares fit to calculate the best transformation between them. Neither of these methods takes into account effects such as possible pixel locking induced by overexpose of particles or the fact that the transverse position of the particles inside the laser sheet induces a parallax between the two camera views. Also, we did not take into account a possible rotation of the cameras in the third dimension. These problems lead to errors in the determination of the particle position and thus a less precise calculation of the transformation parameters. Another potential problem in calculating accurate particle positions would arise if the particles were moving fast with respect to the exposure time of the images, leading to a recording of elongated streaks instead of circular or oval particle images, which might even overlap. Under those conditions, we suppose that the method based on mutual information would produce better results than that based on particle positions. In addition, the subpixel interpolation necessary for creating the joint image might introduce artifacts [40]. In the future, a more precise transformation could be determined by using an image sequence where the laser sheet scans across the system. Then, the particle coordinates can be found in three dimensions, eliminating the problem of parallax.

We therefore recommend the following experimental improvements for setups where this is possible: the laser thickness and illumination should be uniform, overexposure of particles in the overlap region should be avoided, the sensor sensitivity of the two cameras should be equal, and the laser sheet should be thinner than the interparticle distance. Both cameras should be mounted parallel to each other.

For our image sequence, the method based on particle positions performed slightly better than that based on the mutual information. Therefore, we recommend the following algorithm to join the sequence of image pairs for the special case of PK-4 data:

1. Detect all particle positions in the overlap region for both cameras.
2. Find the approximate translation as the median (over the whole sequence) of the particle distances in the image pair-wise overlap region, as described in Section 5, and use this translation to associate particles detected in Camera 1 and Camera 2 with each other. Remove coordinates where no partner was found.
3. Use the particle pairs to find the exact parameters of an affine transformation by least squares fit to the transformation formula (Equation (5)).

Our findings suggest that the image registration for images showing particles works already quite well using comparatively simple least squares fit methods based on the previously detected particle positions.

## Supplementary Materials

The following are available online at https://www.mdpi.com/2313-433X/5/3/39/s1, Video pt_rb: Color coded movie with the right images transformed according to Method II (based on particle positions). Video se_rb: Color coded movie with the right images transformed according to Method I (based on mutual information).

## Abbreviations

The following abbreviations are used in this manuscript:

DC	Direct current
FoV	Field of view
ISS	International Space Station
PK-4	Plasmakristall-4
PO camera	Particle observation camera
